# Successful treatment of cholesterol crystal embolism with anti-proprotein convertase subtilisin/kexin type 9 (PCSK9) antibody: a case report

**DOI:** 10.1080/0886022X.2020.1726383

**Published:** 2020-02-11

**Authors:** Junki Morino, Keiji Hirai, Shohei Kaneko, Saori Minato, Katsunori Yanai, Yuko Mutsuyoshi, Hiroki Ishii, Momoko Matsuyama, Taisuke Kitano, Mitsutoshi Shindo, Akinori Aomatsu, Haruhisa Miyazawa, Kiyonori Ito, Yuichiro Ueda, Susumu Ookawara, Yoshiyuki Morishita

**Affiliations:** Division of Nephrology, First Department of Integrated Medicine, Saitama Medical Center, Jichi Medical University, Saitama, Japan

**Keywords:** Cholesterol crystal embolism, evolocumab, Anti-PCSK9 antibody

## Abstract

**Background:**

We report a unique case of renal cholesterol crystal embolism (CCE) induced by carotid artery stenting that was successfully treated with evolocumab, a fully human monoclonal antibody against proprotein convertase subtilisin kexin type 9 (PCSK9).

**Case presentation:**

A 77-year-old man with hypertension, hyperlipidemia, and chronic kidney disease was referred to our department for decreased estimated glomerular filtration rate (eGFR)—from 32.0 to 13.9 mL/min/1.73 m^2^—5 weeks after carotid artery stenting. Further examination revealed livedo reticularis in the bilateral toes and eosinophilia (723/μL). Skin biopsy from livedo reticularis tissue in the bilateral toes showed cholesterol clefts in the small arteries. The patient was therefore diagnosed with CCE. After 25 weeks’ administration of evolocumab at a dose of 140 mg subcutaneously administered every 2 weeks, his eGFR had improved from 10.7 to 18.1 mL/min/1.73 m^2^.

**Conclusion:**

Evolocumab may have a beneficial effect on renal involvement in patients with CCE.

## Introduction

Cholesterol crystal embolism (CCE) is a systemic disease caused by embolization of cholesterol crystals released from atherosclerotic plaques. The kidneys have been reported to be the most frequently targeted organ of CCE, and approximately 70% of CCE patients develop renal CCEs during their clinical course [1]. Several therapies, such as corticosteroids and low-density lipoprotein (LDL) apheresis, are reported to improve renal outcomes in patients with CCE [2,3]. Steroid therapy, however, has several adverse effects, including gastrointestinal bleeding, hyperglycemia, infections, osteoporosis, and cardiovascular events [4]. LDL-apheresis is invasive because it requires use of an extracorporeal circulation device similar to dialysis [5].

Evolocumab is a human monoclonal antibody that targets proprotein convertase subtilisin/kexin type 9 (PCSK9). It markedly reduces the LDL-cholesterol level [6] through suppression of LDL-cholesterol receptor degradation and subsequent promotion of LDL-cholesterol uptake into the liver *via* the LDL-cholesterol receptor [7]. Evolocumab has also been shown to reduce inflammatory cytokines [8] and promote atherosclerotic plaque regression [9]. Although these findings suggest that evolocumab might have beneficial effects on CCE, its efficacy in that regard has not yet been established. We herein report a unique case of renal CCE induced by carotid artery stenting that was successfully treated with evolocumab.

## Case presentation

**Figure 1.**
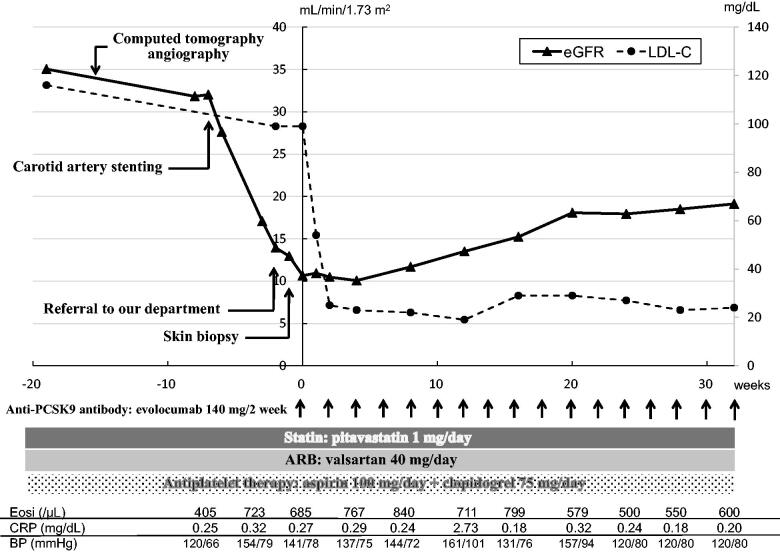
Clinical course of this case. The horizontal axis shows the number of weeks from the initiation of evolocumab administration. The vertical axes show the eGFR and LDL-C levels. ARB: Angiotensin II receptor blocker; BP: blood pressure; CRP: C-reactive protein; eGFR: estimated glomerular filtration rate; Eosi: eosinophils; LDL-C: low-density lipoprotein cholesterol; PCSK9: proprotein convertase subtilisin/kexin type 9.

The patient was a 77-year-old man who had been treated for hypertension, hyperlipidemia, and chronic kidney disease with valsartan 40 mg/day and pitavastatin 1 mg/day. His renal function had been stable, with the serum creatinine around 1.5 mg/dL, estimated glomerular filtration rate (eGFR) 35 mL/min/1.73 m^2^ as calculated by a modified version of the Modification of Diet in Renal Disease formula of the Japanese Society of Nephrology [10], and no proteinuria. Three months before referral to our department, severe right internal carotid artery stenosis was detected by magnetic resonance angiography which was performed to explore reasons for dizziness. Dual antiplatelet therapy with aspirin 100 mg/day and clopidogrel 75 mg/day was initiated. Because right internal carotid artery stenosis of 90% had been shown by computed tomography (CT) angiography 14 weeks before referral to our department, he had undergone carotid artery stent placement 5 weeks before referral to our department. At that time, his eGFR level was 32.0 mL/min/1.73 m^2^. At the follow-up examination 4 weeks after carotid artery stenting, his renal function worsened (eGFR 17.1 mL/min/1.73 m^2^). He was therefore referred to our department for further examination and treatment of the deteriorating renal function (Figure 1).

**Figure 2.**
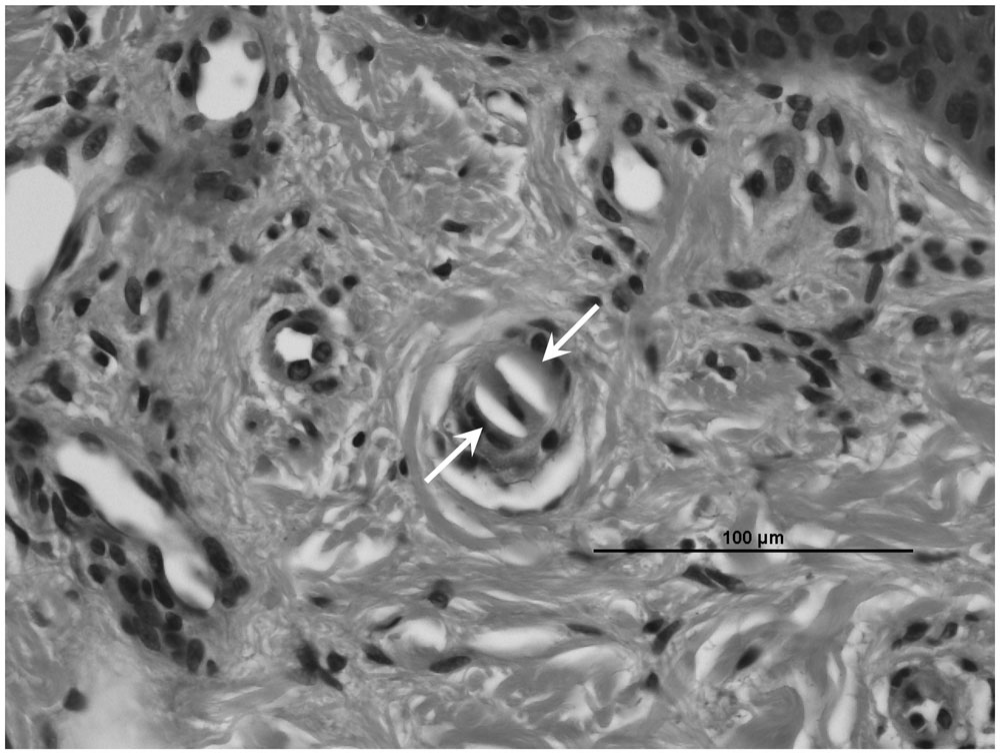
Skin biopsy shows cholesterol clefts (arrows) in a small artery (hematoxylin–eosin stain, ×400).

His systolic/diastolic blood pressure was 141/71 mmHg. Physical examination revealed livedo reticularis in the bilateral toes, with both dorsal pedal arteries palpable. There were no finding of numbness or pain in his extremities. Neither neurological symptoms, such as paralysis, dysarthria, and sensory disturbance nor gastrointestinal symptoms including abdominal pain and gastrointestinal bleeding was observed. Laboratory data showed eosinophilia (723/μL), slightly elevated C-reactive protein (0.32 mg/dL), and severe renal dysfunction (eGFR 13.9 mL/min/1.73 m^2^). Results of serological tests for anti-neutrophil cytoplasmic antibody, anti-glomerular basement membrane antibody, and antinuclear antibody were negative. Serum complement concentrations, including C3, C4, and CH50, were within the normal range. His LDL-cholesterol level was managed at 99 mg/dL under statin administration (pitavastatin 1 mg/day) (Table 1). He had not been taking any medicines that could induce acute kidney injury (e.g., chinese herbal medicine, supplements, analgesics). Renal doppler sonography showed no accelerated blood flow in the renal arteries. Ocular fundus examination showed no evidence of CCE in the retina such as retinal cholesterol crystal emboli. Subsequent skin biopsy specimens from an affected toe revealed cholesterol clefts in the small arteries (Figure 2). Renal biopsy was not performed due to severe bleeding risk because he had been receiving dual antiplatelet therapy to prevent stent thrombosis following internal carotid artery stenting. His clinical and pathological findings pointed to a diagnosis of CCE. He was also considered to be at high risk of atherosclerotic cardiovascular events because he had hyperlipidemia with chronic kidney disease and severe carotid artery stenosis. Hence, evolocumab was administered to reduce and stabilize the aortic atherosclerotic plaque with the expectation that it might improve organ involvement in the CCE (Figure 1). One week later, his LDL-cholesterol level had decreased to 54 mg/dL, and his declining renal function was halted. Evolocumab administration was continued every 2 weeks. The livedo reticularis was alleviated in the bilateral toes. Finally, 20 weeks after the initiation of evolocumab administration, the patient’s renal function, which had gradually improved, plateaued at 18.1 mL/min/1.73 m^2^ (Figure 1).

**Table 1.** Patient’s laboratory results at the time of referral to our department.

Examination	Patient’s level	Reference range
Blood tests		
White blood cells (/μL)	8610	3900–9800
Neutrophils (%)	57.5	40–74
Lymphocytes (%)	21.5	19–48
Monocytes (%)	9.9	3.4–9.0
Eosinophils (%)	8.4	0–7
Basophils (%)	0.4	0–2
Red blood cells (/μL)	394 × 10^4^	427–570 × 10^4^
Hemoglobin (g/dL)	12.1	12.0–17.6
Hematocrit (%)	36.2	39.8–51.8
Mean corpuscular volume	91.9	83–101
Platelets (×10^4^/μL)	35.9	13.0–36.9
Total protein (g/dL)	8.3	6.4–8.2
Albumin (g/dL)	4.6	3.9–5.1
Total bilirubin (mg/dL)	0.53	0.2–1.0
Aspartate aminotransferase (IU/L)	13	11–30
Alanine aminotransferase (IU/L)	10	4–30
Lactate dehydrogenase (IU/L)	210	110–220
Creatine phosphokinase (IU/L)	80	30–190
Total cholesterol (mg/dL)	164	142–248
LDL-cholesterol (mg/dL)	99	<140
HDL-cholesterol (mg/dL)	42	48–103
Triglycerides (mg/dL)	169	30–117
Sodium (mEq/L)	136	138–145
Potassium (mEq/L)	5.2	3.6–4.8
Chloride (mEq/L)	103	100–110
Calcium (mg/dL)	8.6	8.6–10.1
Phosphate (mg/dL)	4.2	2.7-4.6
Blood urea nitrogen (mg/dL)	38	8–20
Creatinine (mg/dL)	3.55	0.65–1.07
eGFR (mL/min/1.73 m^2^)	13.9	
C-reactive protein (mg/dL)	0.32	<0.20
Blood glucose (mg/dL)	121	70–100
HbA1c (%)	5.7	4.6–6.2
IgG (mg/dL)	1983	870–1700
IgA (mg/dL)	421	110–410
IgM (mg/dL)	139	33–190
C3 (mg/dL)	127	65-135
C4 (mg/dL)	32	13–35
CH50 (U/mL)	56.2	30.0–45.0
Antinuclear antibody	≤40	≤40
PR3-ANCA (IU/mL)	<1.0	<1.0
MPO-ANCA (IU/mL)	<1.0	<1.0
Anti-GBM antibody (IU/mL)	<2.0	<2.0
Urine tests		
pH	5.5	5.0–7.5
Specific gravity	1.009	1.005–1.025
Protein	+/−	–
Glucose	–	–
Red blood cells (/HPF)	1–4	0–4
White blood cells (/HPF)	1–4	0–4
BJP	–	–
M protein	–	–
Proteinuria (g/g Cr)	0.27	<0.15

ANCA: anti-neutrophil cytoplasmic antibody; Anti-GBM: anti-glomerular basement membrane antibody; BJP: Bence–Jones protein; eGFR: estimated glomerular filtration rate; HbA1c: hemoglobin A1c; HDL: high-density lipoprotein; HPF: high-power field; IgA: immunoglobulin A; IgG: immunoglobulin G; IgM: immunoglobulin M; LDL: low-density lipoprotein; MPO: myeloperoxidase; PR-3: proteinase-3.

*Note*. eGFR was calculated using a modified version of the Modification of Diet in Renal Disease formula of the Japanese Society of Nephrology: eGFR (mL/min/1.73 m^2^) = 194 × ^−0.287^ × serum creatinine ^−1.094^ (multiplied by 0.739 for women).

## Discussion and conclusion

CCE is a systemic disease characterized by occlusion of small arteries due to CCEs derived from aortic atheromatous plaques, affecting the skin, subcutaneous tissue, brain, eyes, kidneys, gastrointestinal system, and visceral organs [11]. It most commonly occurs iatrogenically after an intravascular procedure or cardiovascular surgery [11]. The kidneys are particularly frequent target organs for CCE, and renal involvement is observed in approximately 70% of patients exhibiting CCE [12]. Among them, 40–60% progress to end-stage renal disease [13,14]. In our case, the patient developed acute kidney injury after carotid artery stenting. Renal function started to rapidly decline 9 weeks after CT angiography, which is not compatible with the course of contrast-induced nephropathy, because serum creatinine concentration usually rises within 24 h after contrast agent administration, peaks at around 3–5 days, and returns to baseline in 7–10 days in contrast-induced nephropathy [15]. All markers of vasculitis were negative, and there was no history of taking nephrotoxic medicine (e.g., analgesics). No findings of renal artery stenosis were noted. Urinalysis showed no hematuria and mild proteinuria, which were compatible with urinary findings of CCE, because hematuria is not often observed and proteinuria is usually in the non-nephrotic range in renal disease caused by CCE [16]. Hence, renal CCE was the most probable cause of his acute renal injury, although we could not exclude the possibility of other renal disease because we did not perform a renal biopsy.

Statins have been reported to decrease the risk of developing end-stage renal disease in patients with CCE [17]. In this case, however, statin therapy did not prevent progression of renal dysfunction. There is no difference in efficacy and safety among strong statins including pitavastatin which this patient was taking [18]. Therefore, we did not change pitavastatin to another statin. Steroid therapy has been shown to have reno-protective effects on CCE [2], although it also can induce various adverse events such as gastrointestinal bleeding, hyperglycemia, infections, osteoporosis, and cardiovascular events [4]. LDL-apheresis has been shown to improve renal function in CCE patients [3], but it has also been reported to induce several serious adverse events, including bleeding, shock, and allergic reactions. LDL-apheresis also requires extracorporeal circulation with the use of a plasma component separator and a specific LDL adsorption column [5].

Evolocumab, a human monoclonal antibody against PCSK9 protein, is administered subcutaneously once every 2 weeks [19], and it is not associated with significant serious adverse events, as shown in a double-blind clinical trial [20]. Evolocumab is therefore considered safer and less invasive than steroids or LDL-apheresis, and it can reduce the patient’s burden. In our case, the patient had hyperlipidemia with chronic kidney disease and severe carotid artery stenosis, for which evolocumab is indicated [7]. Evolocumab was administered to prevent further progression of his atherosclerosis with the expectation that it might alleviate organ involvement with CCE. We did not prescribe steroids for CCE because of concern about enhancing the atherosclerosis progression [1]. As a result, renal function improved in addition to LDL-cholesterol being significantly reduced.

Several studies have indicated that PCSK9 inhibitors have an anti-inflammatory effect on atherosclerotic plaque [9]. PCSK9 inhibition suppresses the production of inflammatory cytokines in macrophages *via* activation of ApoE receptor 2 [21] and down-regulation of NF-κβ [22]. With CCE, cholesterol emboli lodge in small arteries, inducing infiltration of macrophages to the affected arteries and granuloma formation. This inflammatory reaction contributes to thrombus formation and endothelial proliferation, leading to arterial obstruction. Finally, these processes result in ischemic damage and infarction [11]. These findings suggest that evolocumab might suppress macrophage activation and inflammatory cytokine production, which would alleviate renal disease caused by CCE. However, in our case, neither C-reactive protein nor eosinophil showed significant change during evolocumab administration. Serum LDL-cholesterol level is positively correlated with plasma viscosity [23]. It has been reported that intense reduction in LDL-cholesterol by aggressive lipid lowering therapy such as statin and LDL-apheresis improved blood viscosity and increased peripheral arterial blood flow in patients with familial hypercholesterolemia [24,25]. In our case, improvement of renal function was observed, following the marked decrease in serum LDL-cholesterol level. These findings suggest that evolocumab might increase renal blood flow through reduction in LDL-cholesterol, which would ameliorate renal function decline caused by CCE.

In CCE, mechanical obstruction of small arteries and glomerular capillaries in the kidneys by cholesterol crystal emboli reduces regional blood flow, which induces renal function decline [11]. Because renin-angiotensin system (RAS) blockers reduce glomerular filtration pressure *via* dilation of the efferent arteriole [26], interruption of RAS blockers may be effective for improvement of renal function. However, reduced regional blood perfusion activates RAS, leading to oxidative stress, apoptosis, inflammation, and fibrosis [27]. In addition, RAS promotes vascular and tissue remodeling, resulting in progression of chronic kidney disease [27]. In our case, valsartan was continued and renal function was improved after administration of evolocumab. These findings suggest that RAS blockers might have a potential benefit for renal outcomes in CCE. Further studies are needed to investigate the effect of RAS blockers on renal disease caused by CCE.

There is no clinical study that investigated the effects of evolocmab in patients with severe renal impairment. Serum PCSK9 concentration has been reported to be negatively correlated with eGFR [28]. However, a randomized phase III clinical trial has showed that LDL-cholesterol lowering effects of evolocmab were similar regardless of baseline serum PCSK9 level [29]. These findings suggest that evolocmab may have similar effects in patients with severe renal impairment. Further studies are needed, however, to confirm the effects of evolocumab in CCE patients with severe renal impairment.

In conclusion, we presented a case of CCE that was successfully treated with evolocumab. Thus, evolocumab may have a beneficial effect on renal involvement in CCE.

## Declarations

### Availability of data and material

The datasets used and/or analyzed during the current study available from the corresponding author on reasonable request.

## Abbreviations

CCE: cholesterol crystal embolism
eGFR: estimated glomerular filtration rate
LDL: low-density lipoprotein
PCSK9: proprotein convertase subtilisin/kexin type 9
RAS: renin-angiotensin system
